# Aseptic meningitis as the initial presentation of *Leptospira borgpetersenii *serovar Tarassovi: two case reports and a literature review

**DOI:** 10.1186/s12879-021-06200-w

**Published:** 2021-05-27

**Authors:** A. G. N. M. K. Bandara, G. Kalaivarny, Nilanka Perera, J. Indrakumar

**Affiliations:** 1 Colombo South Teaching Hospital, Kalubowila, Dehiwala, Sri Lanka; 2 Faculty of Medical Sciences, University of Sri Jayewardenepura, Gangodawila, Nugegoda, Sri Lanka

**Keywords:** Leptospirosis, Neuroleptospirosis, Aseptic meningitis, *Leptospira borgpetersenii* Serovar Tarassovi

## Abstract

**Background:**

Leptospirosis is a zoonotic illness caused by pathogenic spirochetes of the genus *Leptospira*. The disease spectrum ranges from a mild influenza-like presentation to a more serious Weil’s syndrome. Leptospirosis rarely presents as a primary neurological syndrome. We report two cases of *Leptospira borgpetersenii* serovar Tarasssovi presenting as aseptic meningitis in Sri Lanka.

**Case presentation:**

We describe case reports of two patients presenting as symptomatic aseptic meningitis due to neuroleptospirosis. Both patients had significant neurological involvement at presentation in the absence of common clinical features of leptospirosis. These patients were initially managed as bacterial or viral meningitis and leptospirosis was suspected due to a history of exposure to contaminated water. Subsequently, they were diagnosed to have neuroleptospirosis by positive *Leptospira* serology and both patients gained full recovery.

**Conclusion:**

Our report highlights the importance of considering leptospirosis as a differential diagnosis in patients with aseptic meningitis in endemic settings. Obtaining a detailed occupational and recreational history is helpful in diagnosing neuroleptospirosis promptly. We report the association of *Leptospira borgpetersenii* serovar (sv.) Tarassovi (strain *bakeri*) in causing aseptic meningitis, which has not been reported to the best of our knowledge.

**Supplementary Information:**

The online version contains supplementary material available at 10.1186/s12879-021-06200-w.

## Background

Leptospirosis is a zoonotic disease caused by pathogenic spirochetes of the genus *Leptospira* and it is transmitted direct or indirect contact with the urine of infected animals [1]. The disease spectrum ranges from mild anicteric leptospirosis, characterized by fever, headache, myalgia, saw throat and cough to a more serious Weil’s syndrome manifested as jaundice, renal dysfunction, and bleeding diathesis including pulmonary hemorrhage [2]. According to the published literature, neurological manifestations can be seen in 10–15% of patients with leptospirosis (non-serovar specific) [3]. The most common manifestation of neuroleptospirosis is aseptic meningitis and literature reveals that leptospirosis could account for 5–13% of all cases of aseptic meningitis [1]. The prevalence of aseptic meningitis due to leptospirosis in Sri Lanka is not known. Although meningitis is a well-known feature of leptospirosis [1], it rarely presents as a primary neurological syndrome [3]. Herein we report two cases of primary neuroleptospirosis in the form of symptomatic aseptic meningitis in the background of possible leptospirosis exposure.

## Case presentation

### Case 1

A 37-year-old previously healthy man presented due to fever with chills and rigors, arthralgia and myalgia for 5 days duration. Symptoms were associated with headache, photophobia and recurrent episodes of vomiting. He did not report urinary, respiratory or bowel symptoms to suggest a focal infection. His past medical and surgical history was unremarkable but he admitted to bathing in a stream 1 week prior to admission.

He was febrile without pallor or icterus and did not have any rashes or lymphadenopathy. Examination revealed a Glasgow Coma Scale of 15/15 and neck stiffness. Blood pressure and pulse rate were 120/70 mmHg and 88 beats/minute, respectively. The rest of the examination, including nervous system, was unremarkable.

Investigations revealed a neutrophil leukocytosis and a high C-reactive protein (Table 1). Examination of cerebrospinal fluid (CSF) revealed high protein, normal glucose and predominant polymorphonuclear cells. Blood, urine and CSF bacterial cultures and CSF viral studies were negative. He had raised serum creatinine level on admission which normalized during the next 48 h. Non-contrast computerized tomography of the brain, chest radiograph and ultrasound scan of the abdomen were normal.

**Table 1:** Investigation results of the two patients with neuroleptospirosis

Investigation	Case 1	Case 2	Reference range
Full blood count			
Haemoglobin (mg/dL)	13.5	14	13.5–18
White blood cells (× 10^3^/ μL)	14	26	4.00–11.00
Neutrophils (× 10^3^/ μL)	12.51	24.41	2.0–7.5
Lymphocytes (×10^3^/ μL)	1.8	1.5	1.0–4.5
Platelets (×10^3^ /μL)	329	347	150–400
CRP (mg/dL)	284	14	< 6
Serum creatinine (μmol/L)	238	78.6	62–106
Urine full report			
Protein	nil	trace	nil
Pus cells (/hpf)	nil	5–6	nil
Red cells (/hpf)	10–12	2–3	nil
Liver function tests			
Alanine transaminase (U/L)	103	41.6	5–35
Aspartate transaminase (U/L)	40	26.3	5–40
Alkaline phosphatase (U/L)	416	102	30–120
Gamma-glutamyl transferase (U/L)	328	75	0–30
Total bilirubin (μmol/L)	17.2	10.6	5–17
Cerebrospinal fluid analysis			
White blood cells (/μL)	166	64.7	0–5
Polymorphonuclear cells (%)	96	13	0
Mononuclear cells (%)	3.9	87	60–70%
Protein (mg/dL)	52.4	39	22–38
Glucose (mg/dL)	83.6	72.9	60–80% of random blood glucose
Random blood glucose (mg/dL)	126	110	120–140

The patient was managed as bacterial meningitis with intravenous ceftriaxone (2 g, twice daily) intravenous dexamethasone (8 mg, three times daily). Considering the history of high-risk exposure to possible contaminated water, serum leptospirosis microscopic agglutination test (MAT) was performed, yielding a positive titer of 1/5120. The serovar was identified as *Leptospira borgpetersenii* sv. Tarassovi (strain *bakeri*).

### Case 2

A 26-year-old previously healthy man presented due to diffuse headache associated with neck pain, photophobia and recurrent episodes of vomiting. The patient indicated he suffered from high grade fever, headache, arthralgia and myalgia 10 days prior to examination. These symptoms improved within 3 days of taking an antibiotic prescribed by his general practitioner (drug prescription or remaining drugs were not available with the patient). However, fever recurred with symptoms suggestive of meningeal irritation requiring admission. During further questioning, he revealed a history of water rafting 2 weeks prior to the onset of symptoms. Two friends of the patient who participated in the water rafting also developed similar illness with both requiring hospital admission. The patient’s past medical and surgical history was unremarkable.

Patient was ill-looking and febrile without pallor or icterus. Examination did not reveal any skin rashes or lymphadenopathy. His Glasgow Coma Scale was 15/15 on examination and he had neck stiffness. Kernig sign was negative. His blood pressure and pulse rate were 90/60 mmHg and 100 beats/minute, respectively. The rest of the examination including nervous system, was unremarkable.

His laboratory investigations revealed a neutrophil leukocytosis (Table 1). Liver and renal function tests were normal. CSF examination showed lymphocytic pleocytosis with normal protein and glucose levels. Blood, urine and CSF bacterial cultures were negative. Japanese Encephalitis virus IgM and Herpes Simplex virus antibodies in the CSF were negative. Non- contrast computerized tomography of the brain and the chest radiograph were normal. Due to lymphocytic pleocytosis in CSF, the patient was treated for partially treated bacterial or viral meningitis with intravenous ceftriaxone (2 g, twice daily), intravenous acyclovir (500 mg, three times daily) and intravenous dexamethasone (8 mg, four times daily). Following 2 days of treatment, the patient improved markedly. Leptospirosis MAT was performed due to the high-risk exposure to contaminated water, yielding a positive titer of > 1/840. The serovar was identified as *Leptospira borgpetersenii* sv. Tarassovi (strain *bakeri*).

## Discussion and conclusions

Leptospirosis is a zoonotic illness caused by pathogenic spirochetes of the genus *Leptospira*. The pathogenic *Leptospira* spp. include more than 350 serovars belonging to 30 pathogenic and saprophytic serotypes [4]. Although one million infections occur annually in the world with approximately 60,000 deaths, leptospirosis is still under-reported [4]. Leptospirosis is a widespread disease and it is more prevalent in tropical countries primarily due to occupational exposure than developed countries where it is more related to recreational activities such as swimming and water rafting [2].

Leptospirosis is a biphasic illness with an average incubation period of 5–14 days which can range from 2 to 30 days [5]. It consists of an initial leptospiraemic phase (3–7 days) followed by an immune phase (4 to 30 days). Transmission of spirochetes to humans can occur through direct contact with urine, blood and tissues of infected animals. In addition, infection could occur indirectly through mucosal surfaces and damaged skin exposed to contaminated environments such as moist soil in agricultural lands, lakes, streams and rivers. Carriers of the disease include both wild and domestic farm animals such as rodents, cattle, dogs and pigs. Spirochetes colonize the proximal renal tubules of infected reservoirs hosts and are excreted in the urine [5]. According to the history of our patients, the likely source of infection is exposure to contaminated river water.

Neurological manifestations of leptospirosis include encephalitis (altered sensorium, psychosis, seizures, hemiplegia), intracranial bleeds, cerebellitis, movement disorders, myelitis [6], flaccid paraplegias (including Guillain-Barré syndrome-like presentation), mononeuritis, neuralgias, autonomic lability, polymyositis and acute disseminated encephalomyelitis [1]. Despite these varied manifestations, common neurological presentations were altered sensorium, deeply comatose state and acute symptomatic seizures from a study done on 31 patients treated as leptospirosis [7].

Neurological manifestations can be seen in about 10–15% of patients with leptospirosis although signs of meningeal irritation occur in 80% of neuroleptospirosis cases [8]. According to Panicker et al. [1], 40 patients admitted to a general medical ward over a 3-year period with acute neurological disease were found to have leptospirosis. Available reports of neuroleptospirosis are summarized in Table 2. Primary neuroleptospirosis as the initial presentation is uncommon. As such, in the absence of hepatic or renal involvement, a diagnosis of leptospirosis can be delayed.

**Table 2:** Reported cases of neuroleptospirosis

	Reference	Initial clinical presentation	Identified serovar (sv.)
1	Wang et al., 2016 [9] Atypical leptospirosis: an overlooked cause of aseptic meningitis	Persistent diffuse headache, disturbed sleep, unrelenting vomiting and persistent neck stiffness	*L. santarosai* sv. Shermanii
2	Panicker, Mammachan and Jayakumar, 2001 [1] Primary neuroleptospirosis	Fever, headache, and neck stiffness	common serotypes implicated are Caninola, Icterohaemorragiae, and Pamona
3	Özdemir et al., 2008 [10] An discriminated aseptic meningitis case between rickettsia and leptospiral meningitis	Fever, chills, rigors, headache, and disorientation	*Leptospira biflexa* sv. Patoc
4	Bhatt et al., 2018 [8] Uncommon manifestation of leptospirosis: A diagnostic challenge	Fever, diffuse dull-aching headache with generalized body aches and malaise. Worsening of sensorium in form of decreased responsiveness to commands, decreased verbal output and inability to move his limbs.	None
5	Chang et al., 2003 [11] A case of primary neuroleptospirosis	Sudden onset of paraparesis, urinary retention, and numbness below his upper abdomen of 3 weeks duration.	None
6	Mathew et al., 2006 [7] Neuroleptospirosis - Revisited: Experience from a tertiary care neurological centre from South India	Acute fever with chills and rigors, headache and vomiting, altered sensorium, acute symptomatic seizures	None
7	Saeed et al., 2018 [3] First reported case of neuroleptospirosis complicated with Anton’s syndrome	Flu-like illness with fever, myalgia and headache, seizures, reduced consciousness	None
8	Waggoner et al., 2015 [12] Case report: Molecular detection of leptospira in two returned travelers: Higher bacterial load in cerebrospinal fluid versus serum or plasma	Fever with chills, headaches, myalgia, nausea, vomiting, and loose stools. Stiff neck and photophobia.	None
9	Wang et al., 2012 [13] Leptospirosis with transient paraparesis and thrombocytopenia: A case report	Fever, back pain, bilateral leg weakness, cough and general malaise	None

The most common manifestation of neuroleptospirosis is aseptic meningitis which accounts for 5–13% of all cases of aseptic meningitis in one study [1]. Aseptic meningitis is the presence of clinical and laboratory evidence of meningeal inflammation in the absence of positive routine bacterial cultures. Common etiologies include viral, mycobacterial, fungal, spirochetal infections and non-infective causes such as autoimmune conditions, medications and malignancies.

Though leptospirosis is an uncommon cause of aseptic meningitis, it should be considered during evaluation of patients in endemic areas. According to literature, serovars Icterohaemorrhagiae, Pomona, Grippotyphosa, Canicola, Australis [14] and *Leptospira santarasoi* sv. Shermanii [15] have been reported to cause aseptic meningitis. A pure meningitic syndrome as seen in our second patient is rarely reported. Signs of meningeal irritation are uncommon in leptospiraemic phase and the immune phase is characterized by classical symptoms of headache, vomiting and meningeal irritation. *Leptospira* can be isolated from the CSF while cytology and biochemistry of the CSF are normal except raised CSF pressure during the leptospiraemic phase [1]. During the immune phase of infection, immune complexes may lead to raised CSF opening pressure, lymphocytic pleocytosis (less than 500 × 10^6^/l), raised proteins (up to 0.4-3 g/l) and normal glucose levels [1, 9]. Anti-*Leptospira* antibodies may be detected in CSF during this phase [8]. Highly sensitive molecular diagnostics also have been used to detect small amount of leptospiral DNA in CSF, enabling early detection and prompt treatment [7, 16]. In neuroleptospirosis, CSF analysis almost invariably shows lymphocyte predominance in later phases but also may initially show a predominance of polymorphs [17]. There was a marked disparity in the CSF microscopy of our patients. In the first case, a neutrophilic predominance was observed, while CSF from the second case displayed lymphocytic pleocytosis. The possible reason for this difference could be the more acute presentation of the first patient (after 5 days of illness) compared to the second patient (more than 10 days of illness).

Our first patient had predominant neurological symptoms associated with mild hepatorenal involvement. He did not have thrombocytopenia or pulmonary involvement. The second patient had isolated aseptic meningitis without other organ involvement. Both these patients were diagnosed with *Leptospira* meningitis according to clinical symptoms and a positive serum MAT. Modified Faine’s criteria (see Additional file 1) is a scoring system introduced by World Health Organization (WHO) to diagnose leptospirosis in resource poor settings [8]. It is based on the clinical history (part A), epidemiological history (part B) supported by laboratory parameters (part C). A presumptive diagnosis can be made when the score of part A or the total score of part A & B is/are higher than 26. A total score of parts A, B and C exceeding 25 also is considered suggestive of leptospirosis [8]. The calculated scores of both our patients were higher than 25.

We used serum MAT to confirm leptospirosis in our patients. Current or recent infection with *Leptospira* is evidenced by a single MAT titer of > 1:320. The diagnosis is most specific when a > 4-fold rise in titer is found between acute and convalescent phases [5, 18]. In our first case, the initial titer was 1/5120 which rose to a titer of 1/10240 after 2 weeks. In the second case, patient had an initial titer of 1/840 but refused re-testing. In both patients, the predicted infecting serovar was *Leptospira borgpetersenii sv. Tarassovi* (strain *bakeri*), the most common serovar circulating in Sri Lanka [12]. To our knowledge, this serovar has not been reported to cause primary neuroleptospirosis. Further studies to identify the prevalence of leptospirosis as a causative agent in aseptic meningitis are needed.

Treatment modalities for neuroleptospirosis are not well studied. The use of antibiotics in leptospirosis is based on small number of clinical trials (reviewed in [9, 13]) and the effect on reducing mortality is controversial. Penicillin, cephalosporins, doxycycline and chloramphenicol has been recommended for treatment of leptospirosis and both of our patients received a 3rd generation cephalosporin. The role of antibiotics in the immune-mediated aseptic meningitis should be further evaluated. The role of steroids in managing leptospirosis mediated severe complications is controversial and not supported by adequately powered studies [1]. Additional studies on the effectiveness of steroids in *Leptospira* induced aseptic meningitis are required to guide appropriate therapy for these patients. Prognosis of neuroleptospirosis is favorable according to available evidence [1]. However, presence of altered sensorium and seizures at presentation are known to lead to a worse prognosis [1]. Uveitis, deafness and chronic meningitis also are identified as sequelae of the illness [8]. As such, a prompt diagnosis and appropriate management is important to prevent poor outcome.

Although aseptic meningitis is not common as the primary manifestation of leptospirosis, it should be considered as a cause in the presence of a likely history in endemic areas. Both our patients recovered completely without complications due to timely diagnosis and prompt management. History of possible high-risk exposure was the key finding which helped to suspect neuroleptospirosis in our patients as typical organ involvement was absent. The association of *Leptospira borgpetersenii* sv. and neuroleptospirosis deserves further evaluation.

## Supplementary Information

**Additional file 1.** Modified Faine’s criteria. Modified Faine’s criteria is a scoring system introduced by World Health Organization (WHO) to diagnose leptospirosis in resource poor settings.

## Data Availability

All data gathered during this study are included in this manuscript.

## Abbreviations

CRP: C Reactive Protein
CSF: Cerebrospinal fluid
MAT: Microscopic agglutination test
