# Supplementary material for: Aseptic meningitis as the initial presentation of Leptospira borgpetersenii serovar Tarassovi: two case reports and a literature review
Source: BMC Infect Dis. 2021 May 27;21:488. doi: 10.1186/s12879-021-06200-w (PMC8161910; doi:10.1186/s12879-021-06200-w)
Supplement: Supplementary file 1 — Additional file 1. Modified Faine’s criteria. Modified Faine’s criteria is a scoring system introduced by World Health Organization (WHO) to diagnose leptospirosis in resource poor settings. [file 12879_2021_6200_MOESM1_ESM.docx]

Additional File 1

Table 1: Modified Faine’s criteria

| **Part A: Clinical data** | Score |
| --- | --- |
| - Headache | 2 |
| - Fever | 2 |
| - Fever >39 °C | 2 |
| - Conjunctival suffusion | 4 |
| - Meningism | 4 |
| - Myalgia | 4 |
| - Conjunctival suffusion + Meningism + Myalgia | 10 |
| - Jaundice | 1 |
| - Albuminuria / Nitrogen retention | 2 |
| - Haemoptysis / dyspnoea | 2 |
| **Part B: Epidemiological factors** | Score |
| - Rainfall | 5 |
| - Contact with contaminated environment | 4 |
| - Animal contact | 1 |
| **Part C: Bacteriological and Laboratory Findings** | Score |
| *Isolation of Leptospira in culture – Diagnosis certain* |  |
| - Polymerase Chain Reaction (PCR) | 25 |
| Positive serology |  |
| - Elisa IgM positive* | 15 |
| - SAT positive* | 15 |
| - Other rapid tests** | 15 |
| - MAT – single positive in high titer* | 15 |
| - MAT – Rising titer / seroconversion (paired sera) | 25 |
|  |  |
| **Presumptive diagnosis of leptospirosis is made of:**   - Part A or Part A and Part B score: 26 or more - Parts A, B, C (Total): 25 or more - A score between 20 and 25 suggests leptospirosis as a possible diagnosis. | |

* Any one of the tests only should be scored.

** Other rapid tests - Latex agglutination test / Lepto dipstick / Lepto Tek lateral flow / Lepto Tek Dri-Dot test

Source: Kumar SS. In: Indian Guidelines for the Diagnosis and Management of Human Leptospirosis. Muruganathan A, editor. India: Medicine Update; 2013. pp. 23–29
